# Supplementary material for: Postweaning Development Influences Endogenous VPAC1 Modulation of LTP Induced by Theta-Burst Stimulation: A Link to Maturation of the Hippocampal GABAergic System
Source: Biomolecules. 2024 Mar 20;14(3):379. doi: 10.3390/biom14030379 (PMC10968312; doi:10.3390/biom14030379)
Supplement: Supplementary file 1 [file biomolecules-14-00379-s001.zip › biomolecules-2908441-supplementary.pdf]

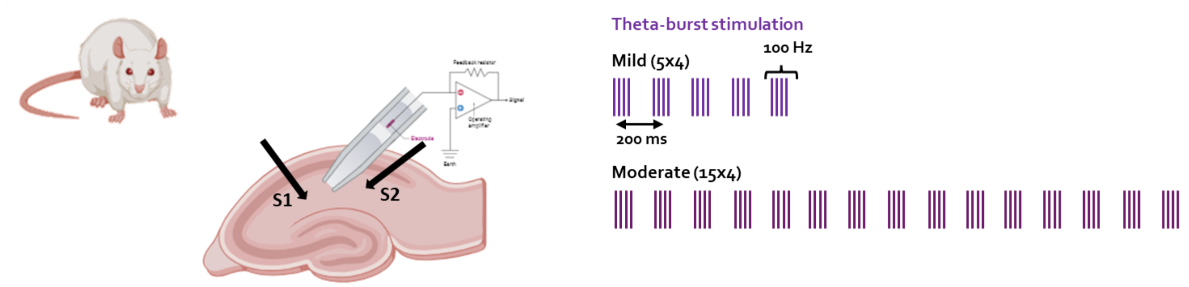


**Figure S1 – Electrophysiological recordings, LTP induction and drug testing. A.** Schematic representation of an hippocampal transverse slice preparation showing the recording configuration used to obtain extracellular responses in the CA1 *stratum radiatum* evoked by stimulation of two separate sets of Schaffer collateral/commissural fibres (S1 or S2) and the mild and moderate TBS patterns used to elicit LTP in rat hippocampal slices.
